# Emerging and Re-emerging Pathogens Causing Blood Stream Infections (BSI) in Hospitalized Patients at a Tertiary Care Hospital

**DOI:** 10.7759/cureus.82587

**Published:** 2025-04-19

**Authors:** Shubhransu Patro, Smrutisree Mohapatra, Arushi Choudhary, Ipsa Mohapatro, Dipti Pattnaik, Basanti K Pathi

**Affiliations:** 1 General Medicine, Kalinga Institute of Medical Sciences, Kalinga Institute of Industrial Technology (KIIT) Deemed to be University, Bhubaneswar, IND; 2 Microbiology, Kalinga Institute of Medical Sciences, Kalinga Institute of Industrial Technology (KIIT) Deemed to be University, Bhubaneswar, IND; 3 Preventive and Social Medicine, Kalinga Institute of Medical Sciences, Kalinga Institute of Industrial Technology (KIIT) Deemed to be University, Bhubaneshwar, IND

**Keywords:** antimicrobial, automation, blood stream infection(bsi), emerging/re-emerging pathogens, paired blood culture

## Abstract

Background and objectives

Infectious diseases known as bloodstream infections (BSIs) are characterized by the presence of live microorganisms in the bloodstream, which are subsequently confirmed by the positive results of one or more blood cultures. These conditions cause or have caused an inflammatory response, which is reflected in changes to hemodynamic, laboratory, and clinical parameters. In the current scenario, hospitalized patients are more likely to die from BSI caused by a number of emerging and re-emerging (E&Re) bacteria. Re-emerging means recurrence or new outbreaks of old infectious diseases with important public health relevance. It is difficult to identify these species using conventional identification methods. Automated techniques can be used for identification and testing for antibiotic susceptibility. The objective of this research is to ascertain the frequency of newly identified E&Re bacterial pathogens that cause BSIs, as well as their pattern of antibiotic susceptibility, risk factors, and post-BSI outcomes.

Materials and methods

This was a retrospective study, carried out over the course of two years (June 2022 to May 2024) at the Department of Microbiology, Kalinga Institute of Medical Sciences (KIMS), Bhubaneswar. All the relevant data were collected from electronic records and entered into an Excel sheet for analysis. An identification/sensitivity testing system (VITEK 2, bioMérieux, Marcy-l'Étoile, France) and automated blood culture (BacT/Alert 3D, bioMérieux, Marcy-l'Étoile, France) were used to process the samples.

Results

In total, 5730 (18%) of the 31850 blood cultures showed positive microbial growth. Paired bottle culture, which came to be positive, was 2718 (47.43%). A total of 430 (15.82%) E&Re pathogens and 2288 (84.17%) common pathogens were isolated from the paired positive blood culture, showing their strong association with BSI. Out of the total E&Re pathogens, bacterial pathogens isolated were 363 (84.41%), and the fungal pathogen (*Candida auris*) isolated was 67 (15.58%). The predominant bacterial pathogen isolated was *Burkholderia cepacia* 126 (29.30%), followed by *Sphingomonas paucimobilis *66 (15.34%) and *Elizabethkingia meningoseptica *54 (12.55%)*. Burkholderia cepacia *shows maximum susceptibility to cotrimoxazole and minocycline.

Conclusion

For optimum isolation, identification, and treatment of BSI pathogens, paired blood culture should be sent to the microbiology laboratory along with the clinical and antibiotic history. These E&Re pathogens in blood culture should be considered as possible infectious agents and should not be ignored as probable contaminants. Early and precise management of patients with BSI is made possible by automation, which aids in the timely reporting of E&Re infections.

## Introduction

The presence of viable bacterial or fungal microorganisms in the bloodstream, as later confirmed by the positive results of one or more blood cultures, that cause or have caused an inflammatory response characterized by changes in clinical, laboratory, and hemodynamic parameters is what defines bloodstream infections (BSIs), which are infectious diseases [1]. With a 30% fatality rate, it results in substantial healthcare expenses and mortality [2]. Emerging infectious diseases are illnesses that are believed to be a serious microbiologic public health concern because they are either newly discovered in people or are spreading quickly in terms of incidence or geographic dispersion. Treatment of nosocomial BSIs is difficult due to the development of multidrug resistance in emerging and re-emerging (E&Re) bacterial pathogens [3]. With their high intrinsic antibiotic resistance, these bacteria cause a considerable amount of morbidity and mortality among hospitalized patients [4].

*Stenotrophomonas maltophilia, Burkholderia cepacia *complex, *Aeromonas hydrophila, Sphingomonas paucimobilis, Elizabethkingia meningoseptica*, and *Ralstonia mannitolilytica* are among the species that belong to this diverse group. Most of them are nosocomial microorganisms that give immunocompromised people opportunistic illnesses [5]. Natural barriers are disrupted because of their invasion and colonization of the sterile site. In a clinical microbiology laboratory, these species make up around 15% of all bacterial isolates [6].

In the past, identification was based on traditional culture-based methods [7] and biochemical testing, which frequently lacked the sensitivity required for quick and precise E&Re pathogen detection [8]. Automation technologies, on the other hand, have shown a promising role as identification tools due to their increased speed, higher throughput, and consistent findings [9].

Significant factors contributing to the rise of E&Re pathogen-induced BSIs in low- or middle-income nations include the absence of standard antimicrobial guidelines, the development of antibiotic resistance, a lack of adequate diagnostic equipment, an unfavorable hospital environment, and the standard of hand hygiene [10]. Therefore, to reduce the mortality and morbidity brought on by these organisms in hospitalized patients, early and accurate detection using automation techniques combined with the right therapy is required [2].

The prevalence of E&Re pathogens and their antibiogram has not yet been reported in various parts of India. This study aimed to isolate and identify the E&Re bacterial pathogens causing BSI from paired blood specimens, ascertain their antimicrobial susceptibility pattern, and find the associated risk factors and clinical outcomes following BSI.

## Materials and methods

Study setting and design

This study was a prospective observational study, carried out in the Department of Microbiology at the Kalinga Institute of Medical Sciences in Bhubaneswar for two years, from 1st June 2022 to 31st May 2024. In the microbiology laboratory, 3,850 samples that were obtained from hospitalized patients were processed.

Furthermore, the study received ethical approval from the Institutional Ethical Committee (KIIT/KIMS/IEC/1981/2025). The study included patients of all ages who had paired bottle positive E&Re bacteria and showed signs of symptoms of septicemia and BSI. However, isolates other than E&Re pathogens, single-bottle positive, and non-septicemic individuals were not included.

Study procedure

Sample Collection and Transport

As a routine procedure of sample collection followed in the hospital, following aseptic precautions and site washing with 70% isopropyl alcohol, about 5-10 mL of blood from adults and 1-5 mL from pediatric patients is collected in blood culture bottles (BacT/Alert 3D, bioMérieux, Marcy-l'Étoile, France) at the patient's bedside. Bottles are then submitted right away for culture in the microbiology laboratory. An identification/sensitivity testing system (VITEK 2, bioMérieux, Marcy-l'Étoile, France) and automated blood culture (BacT/Alert 3D) were used to process the samples, and the results were interpreted in accordance with the Clinical and Laboratory Standards Institute (CLSI) 2022 criteria [11]. The samples collected and transported by this method are tested in the laboratory. Our study sample consisted of blood samples received in the laboratory, which were screened and later included in the study analysis based on the inclusion and exclusion criteria.

Sample Processing

The blood culture vial was immediately put into the BacT/Alert 3D, a completely automated blood culture system for detecting any growth in the blood culture bottle, as soon as it arrived at the lab. Following the bottles' positive flag, a Gram stain was performed, and then the bacteria were isolated by aerobic incubation at 37 °C for 18 to 24 hours, followed by subculturing on chocolate agar, 5% sheep blood agar, and MacConkey agar plates. Colony morphology, Gram stain results, and enzymatic assays (catalase, coagulase, and oxidase) were used for the preliminary identification. The final identification and antimicrobial susceptibility testing were carried out using an automated VITEK 2 system in accordance with the manufacturer's instructions.

Gram Staining

When a blood culture is flagged as positive by the BacT/Alert 3D system, indicating microorganism growth, a Gram stain is performed to confirm the presence of the Gram-positive or Gram-negative bacteria. Blood from the culture bottle is smeared over a clean, sterile glass slide, stained with primary stain, mordant, decolorizer, and counterstain, dried, and viewed under an oil immersion objective. This helps in the early identification of pathogens and gives a presumptive result to start early empirical therapy in critically ill BSI patients.

Culture and Antimicrobial Susceptibility Testing

Blood from the flagged positive bottle was inoculated on blood agar and MacConkey agar with a sterile loop and incubated in a 5% CO₂ incubator overnight. After getting pure isolated colonies (one or more morphotypes), the inoculum was prepared in saline suspension to match the 0.5 McFarland standard and processed for automated identification and antimicrobial susceptibility testing (AST) by VITEK 2 by micro broth dilution, and the result is interpreted in terms of minimum inhibitory concentration (MIC).

## Results

Out of 31850 blood samples, 5730 (18%) were flagged positive for microbial growth. Paired bottle positive was 2718 (47.43%). A total of 430 (15.82%) E&Re pathogens and 2288 (84.17%) common pathogens were isolated from the paired positive blood culture, showing their strong association with BSI. A common pathogen is said to be an organism that can cause disease or illness in its host. Out of the total E&Re pathogens, bacterial pathogens isolated were 363 (84.41%), and the fungal pathogen (*Candida auris*) isolated was 67 (15.58%). Of these isolates, 278 (64.65%) were from male patients, and 152 (35.34%) were from female patients. The predominant bacterial pathogen isolated was *Burkholderia cepacia* (126, 29.30%), followed by *Sphingomonas paucimobilis (*66, 15.34%) and *Elizabethkingia meningoseptica* (54, 12.55%). Other E&Re pathogens were S*tenotrophomonas *maltophilia (22, 5.11%), *Chryseobacterium indologenes* (29, 6.74%), *Aeromonas spp.* (18, 4.18%), *Rhizobium radiobacter* (28, 6.51%), and *Ralstonia spp.* (20, 4.65%) (Table 1).

**Table 1:** Percentage distribution of various emerging and re-emerging bacterial isolates isolated from blood culture (n=363)

Bacterial isolates	Number (%)
Burkholderia cepacia	126 (29.30%)
Sphingomonas paucimobilis	66 (15.34%)
Elizabethkingia meningoseptica	54 (12.55%)
Chryesobacterium indologenes	29 (6.74%)
Rhizobium radiobacter	28 (6.51%)
*Stenotrophomonas *maltophilia	22 (5.11%)
Ralstonia spp.	20 (4.65%)
Aeromonas spp.	18 (4.18%)

Among the 363 isolates of E&Re pathogens obtained from hospitalized patients, the maximum isolates were obtained from ICUs (273, 75.20%) and 90 (24.79%) from wards. Out of the 273 isolates obtained from ICUs, 126 (34.71%), 109 (30.02%), and 18 (4.95%) were from the medical intensive care unit (MICU), the surgical intensive care unit (SICU), and the neonatal intensive care unit (NICU), respectively. Out of the 90 isolates obtained from wards, 30 (8.26%) were from nephrology, 22 (6.06%) were from neurology, 26 (7.16%) were from surgery, and 12 (3.3%) were from pediatrics (Table 2).

**Table 2:** Percentage positivity of emerging and re-emerging bacteria isolated from various units of the hospital NICU: neonatal intensive care unit; MICU: medical intensive care unit; SICU: surgical intensive care unit; PICU: pediatric intensive care unit

Unit	Number (%)
MICU	126 (34.71%)
SICU	109 (30.02%)
PICU	20 (5.5%)
NICU	18 (4.95%)
Nephrology ward	30 (8.26%)
Neurology ward	22 (6.06%)
Surgery ward	26 (7.16%)
Pediatric ward	12 (3.3%)

While obtaining the age-wise distribution of E&Re bacterial pathogens, the maximum number of isolates were from patients of more than 80 years, 136 (37.46%), followed by 41-60 years, 97 (26.72%), and 61-80 years, 80 (22.03%). Rest 36 (9.91%) and 14 (3.85%) were obtained from 21-40 years and 0-20 years, respectively (Table 3)

**Table 3:** Age-wise distribution of emerging and re-emerging bacteria

Age (Years)	Number (%)
0-20	14 (3.85%)
21-40	36 (9.91%)
41-60	97 (26.72%)
61-80	80 (22.03%)
>80	136 (37.46%)

The antibiotic susceptibility test results of the E&Re bacterial pathogens showed the percentage of sensitivity of various antibiotics tested against E&Re isolates identified from blood culture. A high level of resistance was recorded among most of the isolates. Most of the isolates showed good susceptibility to only fluoroquinolones, co-trimoxazole, and minocycline. *Burkholderia cepacia *shows maximum susceptibility to cotrimoxazole and minocycline. *Sphingomonas paucimobilis* was sensitive to cefoperazone + sulbactam, meropenem, cotrimoxazole and piperacillin + tazobactam. *Elizabethkingia meningoseptica* was only sensitive to levofloxacin and minocycline (Table 4).

**Table 4:** Antibacterial sensitivity profile of emerging and re-emerging pathogens CFS: cefoperazone/sulbactam; PIT: piperacillin/tazobacatm; CPM: cefepime; MRP: meropenem; COT: trimethoprim/sulfamethoxazole; AK: amikacin; LE: levofloxacin; CAZ: ceftazidime; MI: minocycline; E&Re: emerging and re-emerging

E&Re bacteria	CFS	PIT	CPM	MRP	COT	AK	LE	CAZ	MI
*Burkholderia cepacia* (N=126)	52 (41%)	8 (6%)	5 (4%)	66 (52%)	87 (69%)	8 (6%)	72 (57%)	69 (55%)	80 (63%)
*Sphingomonas paucimobilis* (N=66)	41 (62%)	38 (58%)	34 51%)	40 (61%)	38 (58%)	42 (64%)	21 (32%)	10 (15%)	19 (29%)
*Elizabethkingia meningoseptica* (N=54)	4 (7%)	0	0	0	20 (37%)	0	49 (91%)	0	51 (94%)
*Chrysobacterium indolgenes* (N=29)	26 (90%)	29 (100%)	29 (100%)	29 (100%)	8 (28%)	29 (100%)	22 (76%)	29 (100%)	0
*Rhizobium radiobacter *(N=28)	15 (54%)	14 (50%)	14 (50%)	14 (50%)	20 (71%)	20 (71%)	20 (71%)	0	20 (71%)
*Strenotrophomonas maltophilia *(N=22)	NA	NA	NA	NA	22 (100%)	NA	22 (100%)	NA	22 (100%)
*Ralstonia spp. (*N=20)	NA	NA	NA	NA	20	20	NA	20	NA
*Aeromonas spp*.(N=18)	15 (83%)	12 (67%)	15 (83%)	8 (44%)	18 (100%)	15 (83%)	18 (100%)	13 (72%)	NA

Following BSI, 71% of patients were discharged in a hemodynamically stable condition, 10% left against medical advice, and death occurred in 19% (Table 5).

**Table 5:** Outcome following bloodstream infections LAMA: leave against medical advice

Outcome	Percentage
Discharge	71%
Death	19%
LAMA	10%

A bivariate analysis taking the chi-square as a test of association was done between BSI-positive with E&Re pathogens and BSI-positive with common pathogens. Categorical variables were taken in row totals and column totals. All age groups have a statistically significant association with the development of BSI, with a p-value of <0.05. Gender has no statistically significant association (Table 6).

**Table 6:** Statistical association between bloodstream infection positivity with common pathogens and emerging and re-emerging pathogens The chi-square test is used to see the association between bloodstream infection (BSI) positivity with common pathogens and emerging and re-emerging (E&Re) pathogens A p-value of <0.05 is considered significant

Variable	BSI positive with E&Re infections (N=430, 15.82%)	BSI positive with common pathogens (N=2288, 84.17%)	Total N=2718 (100%)	P-value
Age group (in years)				
0-20	43 (10%)	540 (23.60%)	583 (21.44%)	<0.0001
21-40	56 (13.02%)	222 (9.70%)	278 (10.22%)	0.046
41-60	117 (27.02%)	752 (32.86%)	869 (31.97%)	0.02
61-80	78 (18.13%)	716 (31.29%)	794 (29.21%)	<0.0001
>80	136 (31.62%)	58 (2.53%)	194 (7.13%)	<0.0001
Gender				
Male	278 (64.65%)	1390 (60.75%)	1668 (61.36%)	0.14
Female	152 (35.34%)	898 (39.24%)	1050 (38.63%)	

Diabetes as a comorbidity has a statistically significant association with the development of BSI, with a p-value of 0.0002. Less than two days of flagged positivity of bottles has a statistically significant association with the development of BSI. Outcome following BSI has a statistically significant association with the development of BSI (Table 7).

**Table 7:** Statistical association between bloodstream infection positivity with common pathogens and emerging and re-emerging pathogens LAMA: leave against medical advice; E&Re: emerging and re-emerging

Variable	BSI positive with E&Re infections (N=430, 15.82%)	BSI positive with common pathogens (N=2288, 84.17%)	Total N=2718 (100%)	P-value
Co-morbidity present				
Hypertension	154 (35.81%)	778 (34.00%)	932 (34.28%)	0.50
Diabetes	160 (37.20%)	642 (28.05%)	802 (29.50%)	0.0002
Length of days for culture positivity				
<=2 days	251 (58.37%)	1626 (71.06%)	1877 (69.05%)	<0.0001
>2 days	179 (41.62%)	662 (28.93%)	841 (30.94%)	
Patient outcome				
Discharged	307 (71%)	1414 (61.80%)	1721 (63.31%)	<0.0001
LAMA	42 (10%)	612 (26.74%)	654 (24.06%)	
Death	81 (19%)	262 (11.45%)	343 (12.61%)	

## Discussion

E&Re pathogens are common in the environment and are now understood to be significant opportunistic and healthcare-associated infections [12]. To treat septicemic patients effectively, it is crucial to accurately and quickly identify these bacteria in a clinical microbiology lab and perform antibiotic susceptibility testing. Clinicians and microbiologists face difficulties when dealing with BSIs caused by E&Re infections because laboratories lack the necessary resources to identify them and detect growing antibiotic resistance [6]. Few types of E&Re bacteria are resistant to widely used antibiotics; however, antimicrobial resistance is on the rise. Treatment for these organisms is costly and complex due to their MDR [12]. The goal of the current investigation was to determine the antibiogram of the E&Re bacterial pathogens that cause BSIs and to assess their prevalence, particularly in hospitalized patients.

Blood culture positivity in our study is 18%, which was lower than that of a study conducted by Agarwal K. et al. in which the positivity rate was 25.7%, and higher than that of a study by M. Wajid et al. in which the positivity rate was 12.4% [13,14]. Different geographic locations, population types, epidemiological variations in the etiological agents, and other variables like the amount or quantity of blood culture samples can all contribute to variations in culture-positive rates [15].

The maximum pathogens isolated in our study were from male patients (64.65%); men seem to have a slightly higher risk of BSIs and mortality from BSIs compared to women, though the reasons are complex and not fully understood, involving factors like immune responses and comorbidities. Similar to our study by M. Wajid et al., male patients suffered (52%) [14]. According to a recent analysis of data from the National Hospital Discharge Survey (US), men are more likely than women to experience sepsis, severe sepsis, and septic shock [15]. Most male patients with BSI in our study (37.36%) were above 80 years old. In a study by Guchhait P et al., BSI was more common in the age group of >60 years [16]. The rate of isolation of E&Re pathogens was higher from ICUs (75.20%) followed by wards (24.8%), similar to the findings by Guchhait P et al., in which 83.3% of isolates were from ICUs and 16.7% were from wards [16].

*Burkholderia cepacia *was the predominant pathogen, 126 (29.3%), causing BSI in our study. A similar observation was seen in a study by Guchhait P et al. in which *B. cepacia* was the most common isolate (24.1%) [16]. In our study *B.cepacia *isolates were maximum susceptible to cotrimoxazole (69%) and minocycline (63%), which was in contrary to the findings by Gangaram U et al., in which *B. cepacia* isolates were maximum susceptible to meropenem (89.4%), tigecycline (89.4%), and minocycline (84.2%) [17]. *Sphingomonas paucimobilis* isolates were mostly sensitive to cefoperazone + sulbactam (62%), meropenem (58%), cotrimoxazole (61%), and piperacillin + tazobactam (58%), like the findings by Lin et al. [18]. Despite having a low clinical pathogenicity, *S. paucimobilis* infections can result in septic shock, especially in patients with weakened immune systems. As a result, its significance cannot be overlooked [18].

In our study, the isolation rate of *Elizabethkingia meningoseptica* was 12.5%, which was lower than that of the findings of Sarathi S et al., 29.30% [19]. The isolates were only susceptible to levofloxacin and minocycline and resistant to aminoglycosides and carbapenems, like the finding by Singh S et al. [20]. Hospitalized patients should be made more aware of *Elizabethkingia* as a new pathogen causing BSI by healthcare professionals (HCPs). Strict infection control procedures and adherence to hand hygiene by healthcare professionals are required to reduce the risk of *Elizabethkingia* outbreaks in intensive care units [19].

In our study, diabetes mellitus has a significant association with the development of BSI, like the findings by Lin et al. [20]. In-hospital mortality following BSI in our study was 19%, which was lower than the findings of Guchhait P et al., in which the mortality rate was higher (66%) [16].

This study documents the E&Re bacterial pathogens and AST patterns causing BSI from paired blood specimens. However, this study has some limitations; E&Re bacterial pathogens from other clinical samples, except paired blood specimens, causing infections except BSI, are not documented. Also, this was a single-site study where patients were coming from a limited geographic area.

## Conclusions

The frequency of different E&Re isolates from a tertiary care facility is revealed by this investigation. This study suggests that E&Re bacterial pathogens, which were previously thought to be pollutants, may now be pathogenic septicemic agents in patients who are in severe condition. By incorporating automated identification tools into our laboratory-based surveillance operations, we have been able to investigate a subset of E&Re bacteria that were previously unknown or incorrectly recognized as causing BSI. Due to different sensitivity patterns, early identification is required to optimize patient outcomes. To choose the right antimicrobials for empirical therapy, doctors need to stay informed on the prevalence and pattern of antimicrobial susceptibility of the circulating microorganisms, as the alarming rise in multidrug-resistant organismsMDROs has rendered many antimicrobial medicines ineffective. A strong antibiotic stewardship program and stringent infection control policies are vital in the epoch of escalating antibiotic resistance. To improve antibiotic usage policies, such as antibiotic restriction, combination therapy, antibiotic usage based on standard antimicrobial susceptibility testing, and antibiotic recycling, it is imperative to monitor the epidemiology of BSI. This will help to lower the incidence of BSI and prevent the emergence of resistance. The yield increases with the volume of blood cultured. Since many BSI patients have relatively low blood bacterial or fungal densities, appropriate volume sampling is, in fact, the most crucial factor for the detection of bloodstream microorganisms. Together with the clinical and antibiotic history, paired blood cultures should be sent to the microbiology lab for the best possible isolation, identification, and treatment of BSI pathogens.
